# Niosomal Curcumin Suppresses IL17/IL23 Immunopathogenic Axis in Skin Lesions of Psoriatic Patients: A Pilot Randomized Controlled Trial

**DOI:** 10.3390/life13051076

**Published:** 2023-04-24

**Authors:** Hanieh Kolahdooz, Vahid Khori, Vahid Erfani-Moghadam, Fatemeh Livani, Saeed Mohammadi, Ali Memarian

**Affiliations:** 1 Student Research Committee, Golestan University of Medical Sciences, Gorgan 49341-74515, Iran; 2 Department of Immunology, Faculty of Medicine, Golestan University of Medical Sciences, Gorgan 49341-74515, Iran; 3 Ischemic Disorders Research Center, Golestan University of Medical Sciences, Gorgan 49341-74515, Iran; 4 Medical Cellular and Molecular Research Center, Golestan University of Medical Sciences, Gorgan 49341-74515, Iran; 5 Clinical Research Development Unit (CRDU), Sayyad Shirazi Hospital, Golestan University of Medical Sciences, Gorgan 49341-74515, Iran; 6 Infectious Diseases Research Center, Golestan University of Medical Sciences, Gorgan 49341-74515, Iran; 7 Stem Cell Research Center, Golestan University of Medical Sciences, Gorgan 49341-74515, Iran; 8 Rheumatology Research Center, Golestan University of Medical Sciences, Gorgan 49341-74515, Iran

**Keywords:** curcumin, IL17, IL23, IL22, Ki67, niosome, psoriasis, S100A7, S100A12, TNFα

## Abstract

Psoriasis (PS) is characterized by hyperplasia of epidermis and infiltration of immune cells in the dermis. A negligible susceptibility of hypodermic permeation for local anti-inflammatory remedies is one of the major causes of medication failures. Although curcumin (CUR) has indicated effectiveness in treatment of inflammation, its successful permeation through the stratum corneum is yet a challenging issue. Therefore, niosome (NIO) nanoparticles were used as curcumin carriers to enhance its delivery and anti-inflammatory effects. Curcumin-niosome (CUR-NIO) formulations were constructed by the thin-film-hydration (TFH) technique and were added to hyaluronic acid and Marine-collagen gel-based formulation. Five mild-to-moderate PS patients (18–60 years) with PASI scores < 30 with symmetrical and similar lesions were included in the study. The prepared formulation (CUR 15 µM) was topically administered for 4 weeks on the skin lesions, in comparison to the placebo. Clinical skin manifestations were monitored and skin punches were obtained for further gene expression analyses. There was a significant reduction in redness, scaling, and an apparent improvement in CUR-NIO-treated group in comparison to the placebo-treated counterpart. The gene expression analyses resulted in significantly downregulation of IL17, IL23, IL22, and TNFα, S100A7, S100A12, and Ki67 in CUR-NIO-treated lesions. Consequently, CUR-NIO could provide therapeutic approaches for the patients with mild-to-moderate PS by suppressing the IL17/IL23 immunopathogenic axis.

## 1. Introduction

Psoriasis (PS) is a systemic inflammatory autoimmune disorder described by the formation of skin plaques with an inflammatory, painful or itchy surface, limiting the quality of life among involved patients [1]. These skin rashes form pink to red patches on white (Type 1 and 2 Fitzpatrick skin type) skins with silvery to white scales, while forming brown to dark patches with grey scales on dark (Type 5 and 6 Fitzpatrick skin type) skins [2]. Psoriasis is reported in all age and gender groups, mostly demonstrated in adults between 45–64 years [3]. PS patients may develop a rheumatologic state called “*psoriatic arthritis* (PsA)” which leads to inflamed, painful and swollen joints, mostly in fingertips and spine [4]. According to recent epidemiological studies, PS involves 2–3% of the world’s population [5]. The PsA condition has been reported in 25–30 percent of PS patients, with varying clinical symptoms [6]. 

The diagnosis of PS typically involves laboratory and clinical assessments; however, histological analyses of the lesions reveal an accelerated renewal of the epidermis, characterized by hyperkeratosis, parakeratosis, and acanthosis, as well as vasodilation and lymphocytic infiltration. Additionally, ki67 overexpression has been observed [7,8,9]. 

Although the hyperproliferation of keratinocytes is the most remarkable characteristic of PS, the underlying mechanism of the loss of control is not well studied. Environmental factors, genetic background, and immune responses may be involved in the pathogenesis of PS [10]. Although the immunopathological mechanisms underlying this disease with defective immune responses are not yet fully understood, research has highlighted the significant roles played by immune cells located in the dermis and epidermis, such as dendritic cells (DCs) and T lymphocytes (T cells) [11,12]. Activated DCs produce high levels of TNFα and IL23, boosting the differentiation of naïve T cells into the Th17 cells. IL17 and TNFα activate keratinocytes, cultivating epidermal hyperplasia and recruiting other inflammatory cells, especially neutrophils [12]. TNFα-dependent pathways along with the IL23/IL17 axis represent a cross-talk between innate and adaptive immunity which are known as the most important immunological factors in PS pathogenesis [13,14].

Recent evidence has highlighted the role of keratinocytes, particularly in their production of IL23, in both the onset and progression of chronic PS. [15]. Moreover, both IL17 and IL23 induce IL22 production, by Th22 cells, which is also known as a key factor in PS development [16,17]. IL22 stimulates epidermal keratinocyte proliferation and activation, and induces epithelial cells to produce antimicrobial peptides that are synergistically upregulated on the side of IL17 [18,19,20]. 

S100 family of proteins are small calcium-binding proteins known as inflammatory antimicrobial peptides (AMPs) [21]. In particular, IL17A could induce production of some AMP components including S100A7 (psoriasin) and S100A12 (calgranulin c) from keratinocytes [22,23]. AMPs not only recruit leukocytes such as neutrophils (PMNs), Th17 cells, DCs, and macrophages, [24] but also induce TNFα production [25]. Furthermore, S100A12 could be considered as the most promising biomarker for PS [26]. 

Although several therapeutic approaches are currently prescribed or under investigation for PS, corticosteroid topical therapy is still applied as the first option of treatment for mild-to-moderate PS [27,28]. Corticosteroids not only have several side effects, but also do not prompt satisfactory clinical impacts [29]. Medication alternatives to corticosteroids include methotrexate, cyclosporine, and small molecule biologicals such as adalimumab (Humira), a TNF-alpha-blocking antibody, and brodalumab (Siliq), a human antibody against interleukins (IL12/23 inhibitors, IL17 inhibitors, IL23 inhibitors) [30,31].

Evidence shows that the effects of some topical herbal remedies may contribute considerably to the healing process and reduce inflammation [32]. Natural products that have been associated with some success include aloe vera, omega-3 fatty acids, turmeric (curcumin), and Oregon grape [33]. Curcumin (CUR) is a derivative of Curcuma longa which has therapeutic and immunomodulatory properties [34]. Several clinical investigations showed that CUR could be beneficial against different cancer types [35,36], cardiovascular diseases (CVDs) [37], and inflammatory and skin diseases [38,39]. It efficiently relieves clinical manifestations, reduces the level of inflammatory markers, slows down disease progression, and prevents disease relapse [40]. Although the role of CUR in PS has not been well described, it was studied in human and experimental animal studies [41,42]. Previous findings have shown that CUR has the potential to suppress inflammatory cytokines including IL23 and IL17, which have major roles in PS pathogenesis and chronic inflammation [43].

Despite promising therapeutic properties, the use of CUR might be limited because of low bioavailability and solubility, insufficient pharmacokinetics, and rapid degradation [44,45]. On the other side, there is an evidence suggesting that nanoparticles encapsulation can enhance its delivery and stability [11]. The encapsulation of lipophilic compositions into the nanoemulsion intensifies their infiltration into the deep skin layers for local delivery, and may increase their efficacy [46]. Niosomes, as non-ionic surfactant-based vesicles, are obtained by hydration of single-chain surfactants and could be stable at 10 to 1000 nm in size. They have the ability to preserve both lipophilic and hydrophilic drugs by encapsulating them in an aqueous compartment and distributing them within the bilayer [47]. The application of niosomes as carriers for local drug delivery could improve the effectiveness and safety of some drugs, including CUR [48,49]. The use of niosomes has been reported in the cosmetics industry [50]. Additionally, several investigations have revealed that the widespread application of gel-based hyaluronic acid and marine collagen in nano-drug delivery structures improve the stability, release, and absorption of the drugs via epidermis in the skin lesions [51]. The excellent solubility of hyaluronic acid has led to its development as one of the most remarkable carriers for topical delivery of medications to the skin, especially in combination with the nano-carriers [52].

Our current clinical trial evaluated the IL17/IL23 immunopathogenic axis besides healing process in skin lesions of PS patients who treated by a topical formulation containing curcumin-niosomes (CUR-NIO) within a hyaluronic acid and marine collagen gel.

## 2. Materials and Methods

### 2.1. Preparing the CUR-NIO Formulation

CUR-NIO was constructed by thin-film hydration (TFH) method, as described previously [53,54]. Briefly, accurately-weighed quantities of Tween80 and Squalene (Sigma, St. Louis, MO, USA) were dissolved in 2 mL of chloroform and methanol in a round bottom flask (2:1 ratio). Then, 10 mL of CUR 1 mg/mL in methanol was added to the combination and mixed gently with a magnet stirrer (25 °C for 15 min). The dissolved mixture was subsequently evaporated (45 °C for 20 min), using a rotary evaporator, under vacuum and constant rotation to obtain a thin film. By utilizing the hand-shaking process, the thin film was hydrated with 5 mL of isotonic phosphate buffer saline (PBS: pH 7.4) at room temperature (RT) to form the CUR-NIO suspension. This suspension was sonicated within an ultrasonic water bath for 20 min, purified using a 0.2 µm membrane filter, and kept in −80 °C for 2 h. Finally, it was lyophilized in a vacuum freeze-dryer (Crisp Beta 2-8LD plus, Osterode am Harz, Germany) for 24 h in −50 °C.

### 2.2. Characterizing the CUR-NIO Composition

#### 2.2.1. Size, Distribution and Zeta Potential

The size and distribution of constructed niosomes were specified by measuring the vesicles in each preparation using Dynamic Light Scattering (DLS). The lyophilized nanoparticles were dispersed in PBS (pH 7.4) using an ultrasonic water bath for 5 min at 25 °C and the parameters were evaluated. The zeta potential, size, and polydispersity index (PDI) of each preparation was assessed by DLS (Zetasizer Nano ZS; Malvern Instruments, Malvern, UK) utilizing an argon laser beam at 633 nm and a 90° scattering angle. Each sample was measured in triplicates and results were expressed as means ± standard deviation (SD). 

#### 2.2.2. Encapsulation Efficiency (*EE*%) and Loading Capacity of Vesicles

The encapsulation efficiency (*EE*%) and loading capacity (*LC*) was assessed and quantified by the direct method through the following formulations. We dissolved 1 mg of lyophilized CUR-NIO in 1 mL of methanol and evaluated by UV–VIS spectrophotometry at 425 nm (Shimadzu, Japan). The amount of entrapped CUR was assessed using the CUR standard curve.

$$EE\%=\frac{\mathrm{Amounts}\;\mathrm{of}\;\mathrm{encapculated}\;\mathrm{curcumin}*100}{\mathrm{Initial}\;\mathrm{amount}\;\mathrm{of}\;\mathrm{curcumin}}$$

$$LC\%=\frac{\mathrm{Amounts}\;\mathrm{of}\;\mathrm{encapculated}\;\mathrm{curcumin}*100}{\mathrm{Initial}\;\mathrm{amount}\;\mathrm{of}\;\mathrm{nanoparticle}}$$

#### 2.2.3. Preparing the of CUR-NIO Loaded Topical Cream

Topical formulations should contain adequate viscosity to be suitable for administration to the skin. Hyaluronic acid (0.1%) and marine-collagen (2.5%) (formulated by Kimia Golestan Green Chemical Company, Gorgan, Iran) were selected as the matrix of the CUR-NIO gel. Hyaluronic acid and collagen were added into the lyophilized CUR-NIO in a drop-wise fashion, with gentle, but constant stirring in darkness for 2 h. The concentration of CUR-NIO was 0.1% (*w*/*w*) in the gel.

### 2.3. Patients and Sample Selection 

#### 2.3.1. Criteria

Five patients, aged 18 to 60 years, with mild-to-moderate PS (PASI < 30, based on the PS area severity index [55]) and at least two symmetrical and/or similar skin lesions were enrolled in this study. Patients were not pregnant or breastfeeding and were not taking any kind of corticosteroids or topical treatments in the last two months or during the trial. They were not suffering from metabolic syndrome, hepatic, renal or other autoimmune diseases. The key demographic and clinical characteristics of the psoriasis patients included in the study are presented in Table S1. We also used peripheral blood samples from five healthy controls. Written informed consents were signed by all participants. This study was approved by the committee of research ethics at Golestan University of Medical Sciences (IR.GOUMS.REC.1397.275) and registered in the Iranian Registry of Clinical Trials (IRCT20181217042030N1). 

#### 2.3.2. Ex-Vivo Study

At first, for ex vivo evaluation including cell cytotoxicity and gene expression assessments, we isolated peripheral blood mononuclear cells (PBMCs) from blood samples of healthy donors and patients using Ficoll-Paque density gradient centrifugation, as described [56]. 10^6^ live cells were treated with CUR, CUR-NIO, NIO, and a vehicle control (DMSO), in separate wells for 6 h. They were subsequently stimulated by anti-CD3 (1 µg/mL; Sinabiotech, Tehran, Iran), as described previously [57]. After four days, treated PBMCs were collected for cell apoptosis and gene expression analyses.

#### 2.3.3. Intervention

Instructions on application of the formulation were given to the patients, in which they were instructed to apply a thin layer of the 0.1% CUR-NIO gel and the placebo twice a day for four weeks onto their eligible skin lesions, in the manner of a placebo-controlled clinical trial. Specifically, each patient used CUR-NIO gel for one lesion and placebo for the counterpart. The placebo was only composed of gel-based hyaluronic acid and marine-collagen. After four weeks of treatment, the drug- and placebo-treated psoriatic lesions were assessed by a dermatologist and skin punches were obtained from both lesions of each patient for further gene expression evaluation. 

### 2.4. Apoptosis Assessment of CUR-NIO

Flow cytometry measurements were conducted using FITC-conjugated Annexin V and PI staining kits (BioLegend, San Diego, CA, USA) for apoptosis. Isolated PBMCs from healthy donors were cultured as described above and treated with three concentrations of CUR (5, 10 and 15 mM). After four days, cultured cells were harvested and prepared for assessment following the manufacturer’s directions. Finally, each tube was immediately evaluated using BD accuri C6 flow cytometer (BD PharMingen, San Diego, CA, USA). The data were analyzed using the BD Accuri™ C6 software version 1.0.264.21 (Accuri Cytometers, Ann Arbor, MI, USA).

### 2.5. Quantitative Real-Time PCR

Total RNA was isolated from homogenized samples in RNX-Plus (Sinacolon, Tehran, Iran) for RNA extraction and then reverse transcribed into cDNA using Yekta-Tajhiz cDNA synthesis Kit (Tehran, Iran), according to the manufacturer’s protocols. The primers of genes including IL17A, IL22, IL23, TNF-α, S100A7, S100A12, ki67, and 18sRNA (as internal control) were synthesized by *Takapuzist* Gene Molecular Biotechnology Co. (Tehran, Iran), listed in the Table 1. Real-time qPCR amplifications were performed using Master Mix SYBR green kit (Parstous, Mashhad, Iran), which was used for each mRNA on a Real-time PCR detection system (Bioer Technology, Hangzhou, China). The cycle of threshold (Ct) for every gene and the internal control were determined for each sample. The relative mRNA expression was quantified, using 2^−dct^ method. Each sample was tested in triplicate [58].

### 2.6. Statistical Analyses

Statistical analyses of the obtained data and graphs were prepared using SPSS 16.0 and GraphPad Prism 5.04 statistical software (GraphPad, Boston, MA, USA). One-way ANOVA or the equivalent non-parametric Kruskal–Wallis test was utilized to obtain the differences of means between two groups, while Tukey’s post hoc test was performed to confirm the significant differences. In addition, independent samples *t*-test or Mann–Whitney U test were used to compare the means between two groups. *p*-values smaller than 0.05 were considered to be significant.

## 3. Results

### 3.1. Characterizing NIO Nanoparticles

CUR was effectively encapsulated in niosome particles by the single emulsion solvent evaporation technique. The average diameter of nanoparticles (13 ± 2.20 nm), polydispersity index (PDI) (less than 0.2), mean zeta (ξ) potential (2.40 ± 0.602 mV), encapsulation efficacy (86%), and loading efficiency (7.35%) of these NPs were evaluated.

### 3.2. The CUR-NIO Suspension Exerted Low Toxic Effects on PBMCs

**Figure 1:**
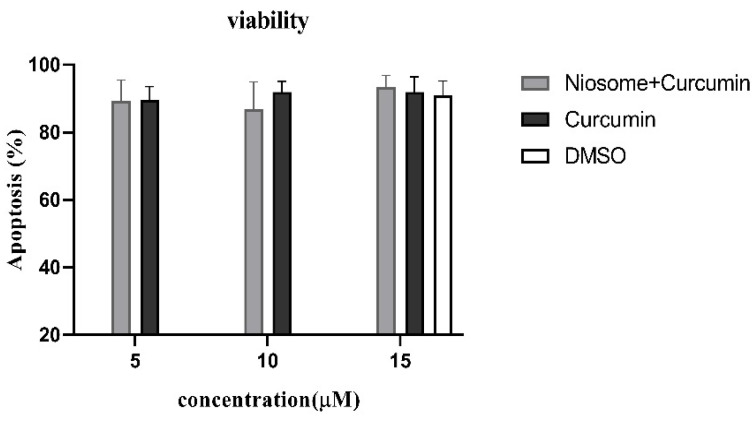
Cytotoxicity assessment by apoptosis. PBMCs from healthy volunteers (*n* = 5) were cultured with CUR, CUR-NIO, and DMSO; curcumin concentrations were 5, 10, and 15 µM. After 4 days, apoptosis was analyzed by flow cytometry.

The apoptosis assay was used to confirm that the CUR-NIO suspension has trace cytotoxicity. PBMCs from healthy donors were treated with CUR, CUR-NIO, NIO and DMSO; CUR had three different concentrations (5, 10, 15 µM) (Figure 1). In all three concentrations the survival rate of the PBMC was almost the same. The highest expected concentration (15 µM) was selected for further analyses. 

### 3.3. Effect of CUR on IL17 Gene Expression in PBMCs from Healthy Donors and PS Patients 

**Figure 2:**
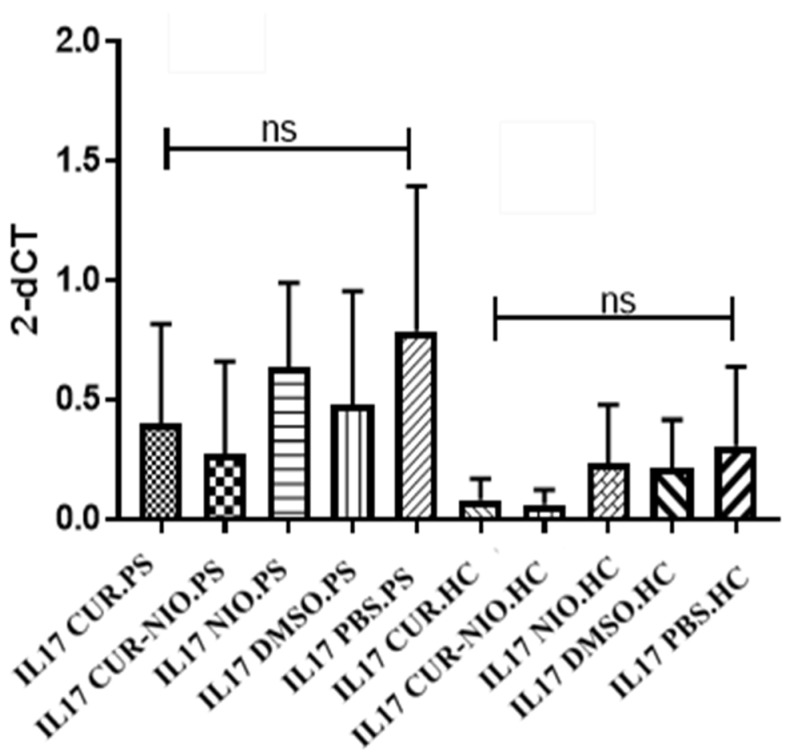
Relative gene expression of IL17 in treated PBMCs with different CUR combinations from PS patients (*n* = 5) and healthy donors (*n* = 5). Although CUR was observed to reduce IL17 gene expression and higher levels of IL17 were detected among psoriasis patients in all treatment groups, our results did not reveal any significant changes across the different treatments in PBMCs from the patient and healthy donor groups. Bars depict relative gene expression of IL17 in stimulated PBMCs by anti-CD3 following treatment with CUR, CUR-NIO, NIO, and DMSO as vehicle control, for 4 days. The results are demonstrated as the mean (±SEM) of the measured gene expression (2^−dCT^). Statistical significance was determined by one-way ANOVA, with Mann Whitney test to compare treatment groups against the control group (ns: non-significance). CUR: curcumin, CUR-NIO: curcumin-niosome, NIO: noisome, PS: psoriasis, HC: healthy control.

Despite the higher expression levels of IL17 among PS patients in all treatment groups and the CUR effect on reducing IL17 gene expression, our results demonstrated no significant change in the PBMCs from patients or from healthy donors for any treatment (Figure 2). Comparisons between patients and normal subjects within similar combinations, however, showed significantly higher levels of IL17 in patients’ cells (*p* < 0.05).

### 3.4. CUR-NIO Gel Reduces Inflammatory Cytokines in Psoriatic Lesions

**Figure 3:**
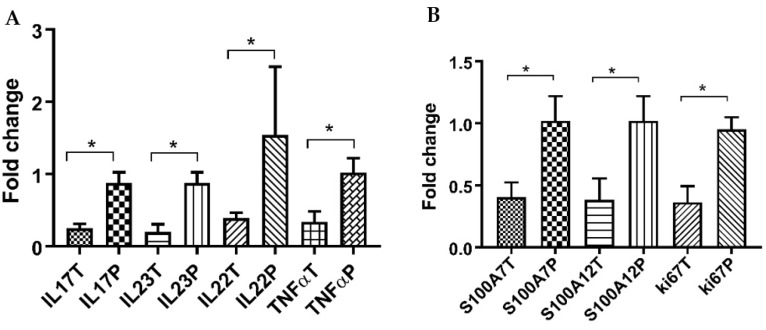
Relative expression of IL17, IL23, IL22 and TNFα genes (**A**) and S100A7, S100A12 and Ki67 genes (**B**) after CUR-NIO gel treated (T) versus placebo (P) in psoriatic skin lesions. mRNA expression was assessed by qPCR in skin tissues from CUR-NIO and placebo groups following 4 weeks of topical administration. The C_t_ values were normalized to 18s RNA. All mRNA expressions were significantly decreased in CUR-NIO group, compared with the placebo. The asterisk marks indicate significant differences, which are shown on the brackets in the figures (* for *p* < 0.05). Data show mean ± SEM.

As demonstrated in Figure 3A, the mRNA expressions of IL17, IL23, IL22, and TNFα in skin lesions of PS patients were significantly decreased following treatment by CUR-NIO gel in comparison to the placebo (*p* < 0.05).

### 3.5. Effects of CUR-NIO Gel on S100A7, S100A12 and ki67

There were also significant downregulations in expression of all S100A7, S100A12, and ki67 genes in skin lesions among CUR-NIO gel treated PS patients compared to the placebo counterparts (*p* < 0.05) (Figure 3B).

### 3.6. Clinical Observation of the Skin of PS Patients

**Figure 4:**
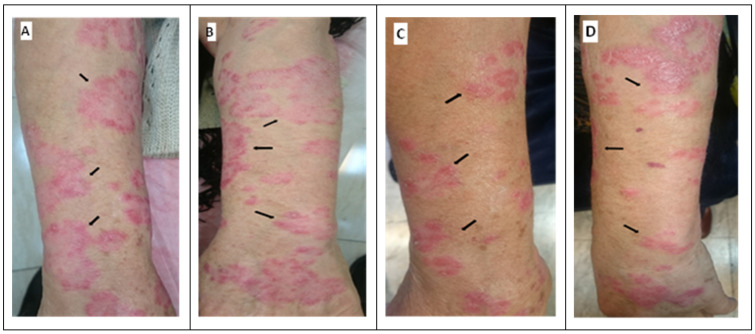
Clinical manifestations of skin psoriasis of a selected patient before and after the application of CUR-NIO gel 0.1% versus placebo. Before application of CUR-NIO gel 0.1% (**A**). Before application of placebo (**B**). After treated with CUR-NIO gel 0.1% (**C**). After treated with placebo (**D**). The skin of a PS patient was examined before and after treatment with CUR-NIO gel compared to a placebo. The results indicated that using the CUR-NIO gel had positive effects on the patients’ lesions, such as a reduction in redness, PS plaque levels, and scaling. Additionally, the uncomfortable symptoms of itching and skin dryness also decreased in lesions following treatment with CUR-NIO gel compared to placebo. Moreover, the redness and inflammation surrounding the skin lesions significantly improved. Representative photographs show localized improvement of psoriasis on the patient’s skin with reduced redness, scaling. The presence of hyaluronic acid and marine-collagen compounds in both pharmaceutical and placebo gels reduced inflammation in both symmetrical lesions. Black arrows indicate intended lesions before (**A**,**B**) and after (**C**,**D**) treatment.

Figure 4 illustrates the skin of a selected PS patient before and after treatment with the CUR-NIO gel versus placebo. Similar to our quantitative evaluation, we observed that employing CUR-NIO gel had positive clinical effects on the patients’ lesions, including reduced redness, levels of PS plaques, and scaling in patients. Furthermore, itching and skin dryness as annoying manifestations of PS declined in lesions following treatment by CUR-NIO gel, in comparison to placebo. Moreover, redness and inflammatory margin in skin lesions were shrunken and considerably ameliorated. 

## 4. Discussion

As an autoimmune disease, PS affects a small population globally but negatively impacts patients’ quality of life. To date, conventional treatments such as topical corticosteroids, phototherapy, and several systemic anti-inflammatory drugs have not been entirely successful in patient treatment and can have various adverse effects [59]. Therefore, achieving effective formulations with the most negligible side effects should be developed for the treatment of PS. Various animal investigations and clinical trials have been conducted to understand the molecular mechanisms of PS pathogenesis better and to introduce novel therapeutic agents. A vast number of medications can effectively control PS; approximately 75% of patients with mild-to-moderate PS respond positively to local treatments [60]. Of note, CUR, the major derivative of curcuma longa, may have anti-inflammatory effects on psoriasis patients [61]. Curcumin has been introduced as a beneficial compound for the management of various inflammatory skin diseases [62]. Kang et al. evaluated the inhibitory effects of CUR on Kv1.3 potassium channels on T cells, and demonstrated the anti-inflammatory roles of CUR by showing that the application of 10 μM of CUR significantly inhibited the secretion of inflammatory factors, such as interleukin (IL)-17, IL-22, IFN-γ, IL-2, IL-8, and TNF-α in T cells. However, their study also revealed that the proliferation of T cells was inhibited by more than 50% when exposed to 100 μM curcumin. It is important to recognize that their study was conducted using a mouse model, which may not be entirely representative of human skin [63]. There have been other studies evaluating the effects of curcumin on psoriasis patients; Kurd et al. administered it orally but did not evaluate inflammatory or immunologic factors [64]. Sarafian et al. did not mention or quantify the exact concentration of the curcumin used in their study [65]. Thus, further research was needed to determine the optimal dose and mode of administration for curcumin in treating psoriasis in humans. Consequently, an examination was conducted to determine the potential toxicity of curcumin on PBMCs at various concentrations. The concentration of 15 μM was identified as having the least toxic effects, and was therefore chosen as the concentration for testing.

Our study found that the positive effects observed on PS patients’ skin lesions after treatment with CUR-NIO gel were supported by a decrease in the expressions of IL17, IL23, IL22, and TNFα in the skin lesions. These results align with previous findings, indicating that CUR-NIO gel has anti-inflammatory properties that can effectively alleviate the symptoms associated with PS. Varma et al. demonstrated that the proliferation of psoriatic-like cells was inhibited, while the apoptosis induction was increased by CUR via downregulation of pro-inflammatory cytokines, such as IL-17, TNFα, IFNγ, IL-6 [66]. Jain et al. showed that the phenotypic and histopathological features of PS skin treated with tacrolimus and CUR-loaded liposphere gel, were enhanced and, the level of TNF-α, IL-17 and IL-22 were reduced compared to imiquimod group [67]. Antiga et al. demonstrated that CUR could be effective as an adjuvant therapy for the treatment of psoriasis vulgaris by reducing serum levels of IL-22 [39]. Sun et al. demonstrated that topical application of CUR encapsulated in nanoparticles in a mice model of PS might help reduce inflammatory cytokines including IL17/IL23, IL22 and TNFα [68]. 

CUR with its anti-inflammatory and antioxidant properties can reduce inflammation. The anti-inflammatory properties of CUR on PS, in addition to its low toxicity, have been defined in various studies [69]. However, the main problem of CUR is its relatively low solubility and low capacity for skin penetration which leads to its rapid disappearance in the epidermis [70]. Algahtani et al. showed that the CUR nanoemugel could be a promising candidate for the long-term management of PS in mice [71]. However, no study, to the best of our knowledge, has studied the effects of CUR-NIO in a clinical fashion on PS patients. Permeation of CUR increases 7–8-fold when formulated with permeation enhancers like nanoparticles and gel form [72]. In the present study, niosome nanoparticles were used to increase the efficiency and absorption of CUR in the psoriatic skin lesions. Niosomes have the capability of modifying the structure of the stratum corneum with the assistance of their surfactant properties and enhancing the smoothness by the substitution of lost skin lipids. They play an important role in drug delivery for a wide variety of dermally active compounds and aid as a safe penetration enhancer [73]. Some studies have shown that the application of niosomes increases the absorption of topical drugs for the treatment of skin diseases, including PS and dermatitis [74,75]. It was found that the niosomes prepared for this study had a mean diameter of about 14 nm, indicating they were nanoparticles of the right size, thus making them easily pass through the stratum corneum and causing DCs to uptake CUR-NIO, thus modulating an immune response [76]. Moreover, hyaluronic acid’s ideal solubility has been considered one of the most important carriers for topical drug delivery to the skin [77]. Therefore, this study considered hyaluronic acid and marine-collagen as the CUR-NIO matrix.

Contrary to our hypothesis, CUR-treated PBMCs of PS patients and healthy donors demonstrated no significant decrease in IL17 gene expression in any of the studied combinations (Figure 2). Brück et al. also showed that the effects of CUR on T cell polarization were not mediated by direct action. Instead, the particular population of immune cells affected by CUR is the DC population [78]. This evidence could support the insignificant decrease in IL17 gene expression observed in our samples. However, another study showed a substantial effect of CUR (at higher concentrations) on inhibition of IFNγ and IL17A-producing cells [79].

As observed by Sun et al., CUR could affect the IL23/IL17 axis in mice by inhibiting IL1β/IL6 and indirectly regulating IL17/IL22 expression [80]. In this regard, CUR-NIO gel significantly decreases the expression of all tested genes related to IL23/IL17 axis (including IL23, IL22, TNFα and IL17) in the psoriatic lesions, compared to the placebo arm (Figure 3). Furthermore, ki67 downregulation following CUR administration in our patients (Figure 3B) could indicate the control of abnormal keratinocyte proliferation in PS skin tissues, which was also observed previously in an in vitro study [81].

We discovered that there were notable reductions in the expression of S100A7, S100A12, and ki67 genes in skin lesions of PS patients treated with CUR-NIO gel, as compared to lesions that were treated with the placebo. The IL17/IL22 could upregulate the S100A7 and S100A12 expression in keratinocytes reciprocally. The keratinocyte-secreted S100A7 may stimulate the production of pro-inflammatory cytokines and recruit immune cells into the skin, which indicates a distinct feature of psoriatic skin lesion [22,23,82]. According to the previous studies, S100A7 and S100A12 are the most promising markers of PS disease activity, and increased expression of both are typical features shown in PS [26,83] and CUR could reduce their expression, as observed in HaCaT keratinocyte cell line [84]. Substantial downregulation of these peptides by CUR-NIO gel in our patients (vs. placebo, Figure 3B) signify the therapeutic capacity of our treatment. 

No adverse effects, such as allergic reactions, skin irritations, staining, or photosensitivity were observed during the studied treatments. The most important limitation of our study was related to the criteria for patient selection, which resulted in a low number of patients with mild/moderate disease severity, no corticosteroid treatment, and at least two symmetrical/similar skin lesions for drug and placebo treatments. However, the last criterion meant that each patient was his/her own control and this was the most important advantage of our study. Consequently, any interference regarding genetic and environmental variations was eliminated which makes the comparisons between treatment and placebo very valuable and reliable in our clinical study.

## 5. Conclusions

In conclusion, the administration of our CUR-NIO gel as a topical drug could stimulate therapeutic effects on the psoriatic skin lesions and lead to healing processes. This could be induced by expression regulation in the main pathogenic inflammatory genes in PS patients. CUR-NIO gel possibly will be a promising drug for improving the quality of life of PS patients.

## Figures and Tables

**Table 1:** The list of primers sequences used in real-time PCR.

	Gene	Plus (5′ > 3′)	Minus (5′ > 3′)	Product (bp)	T_m_
1	IL23A	TCAGGCTCAAAGCAAGTGGA	AGCAGCAACAGCAGCATTAC	128	60
2	IL17A	CGCAATGAGGACCCTGAGAG	TAGTCCACGTTCCCATCAGC	92	60
3	IL22	AGCCCTATATCACCAACCGC	TCTCCCCAATGAGACGAACG	87	60
4	TNFα	CATCCAACCTTCCCAAACGC	CTGTAGGCCCCAGTGAGTTC	246	60
5	S100A7	CACTCAAGCTGAGAGGTCCAT	AAAGACATCGGCGAGGTAATTTG	169	60
6	S100A12	ACCACTGCTGGCTTTTTGCT	GGGTGTCAAAATGCCCCTTC	150	60
7	Ki67	TCTGTTATTGATGAGCCTGTA	GTTGACTTCCTTCCATTCTG	107	58
8	18srRNA	ACCCGTTGAACCCCATTCGTGA	GCCTCACTAAACCATCCAATCGG	159	60

## Data Availability

Data would be available by the corresponding authors upon request.

## Supplementary Materials

The following supporting information can be downloaded at: https://www.mdpi.com/article/10.3390/life13051076/s1, Table S1: Key demographic and clinical characteristics of the psoriasis patients.
