# Supplementary material for: Niosomal Curcumin Suppresses IL17/IL23 Immunopathogenic Axis in Skin Lesions of Psoriatic Patients: A Pilot Randomized Controlled Trial
Source: Life (Basel). 2023 Apr 24;13(5):1076. doi: 10.3390/life13051076 (PMC10224439; doi:10.3390/life13051076)
Supplement: Supplementary file 1 [file life-13-01076-s001.zip › life-2294331-supplementary.pdf]

Supplementary Table S1. Key demographic and clinical characteristics of the psoriasis patients included in the study.

Patient number	Sex	Age	PASI score	Heredity background	Lymphocyte percent in peripheral blood
PS001	Female	52.0	24.0	Yes	41
PS002	Female	46.0	14.0	Yes	49
PS003	Female	48.0	18.0	No	35
PS004	Female	44.0	20.0	Yes	58
PS005	Male	38.0	14.0	No	47
